# Primary Squamous Cell Carcinoma of the Breast: A Case Report and Comprehensive Literature Review

**DOI:** 10.7759/cureus.106178

**Published:** 2026-03-31

**Authors:** Fawad Talat, Hoor Ali, Abdul Subhan Talpur, Obuli Srinivasan Gurunathan, Tzvetan Kozarski, Mansi Kallem, Madhuri Yalamanchili

**Affiliations:** 1 Internal Medicine, United Health Services (UHS) Wilson Medical Center, Johnson City, USA; 2 Pathology, United Health Services (UHS) Wilson Medical Center, Johnson City, USA; 3 Hematology and Oncology, United Health Services (UHS) Wilson Medical Center, Johnson City, USA; 4 Medical Oncology, Broome Oncology, Johnson City, USA

**Keywords:** breast cancer, immunotherapy, metaplastic carcinoma, squamous cell carcinoma, triple-negative breast cancer

## Abstract

Primary squamous cell carcinoma (SCC) of the breast is one of the rarest forms of metaplastic breast carcinoma. It is typically triple-negative, high-grade, and is associated with aggressive clinical behavior. As it is an extremely rare condition, there is no standardized treatment protocol. We present a case of primary SCC of the breast in a postmenopausal woman who presented with a rapidly growing mass of the breast and an inverted nipple. Histopathology confirmed the diagnosis of SCC of the breast, and immunohistochemistry revealed diffuse p40 positivity. The patient underwent bilateral mastectomy and axillary lymph node dissection, followed by adjuvant chemotherapy and immunotherapy. The patient completed chemotherapy, but immunotherapy had to be stopped due to immune-related endocrine toxicity. This case highlights the diagnostic challenges, treatment modalities, and the emerging role of immunotherapy in this rare subtype of breast cancer.

## Introduction

Primary squamous cell carcinoma (SCC) of the breast is an extremely rare form of metaplastic breast cancer and accounts for less than 0.1% of breast malignancies [1]. It is characterized by the predominant presence of malignant squamous cells within the tumor and the absence of an associated primary squamous malignancy elsewhere, and the tumor should not originate from the overlying skin or nipple [1,2]. Clinically, it usually presents as a rapidly enlarging breast mass and/or nipple inversion/discomfort [3]. Due to its rarity, a large portion of the research on this malignancy consists of isolated cases as well as smaller case cohorts. These tumors are normally aggressive, triple-negative, and have an increased likelihood of local recurrence as well as distant metastasis [2,3]. Furthermore, these tumors have an unpredictable response to conventional chemotherapeutic agents typically used in breast malignancies, and the absence of standardized treatment protocols - compounded by limited targeted therapeutic options - makes clinical management particularly challenging [3]. We present a case of PD-L1-high, triple-negative primary SCC of the breast treated with chemo-immunotherapy, complicated by immune-related adverse effects, to contribute to the limited literature on optimal therapeutic strategies for this rare malignancy.

## Case presentation

**Figure 1:**
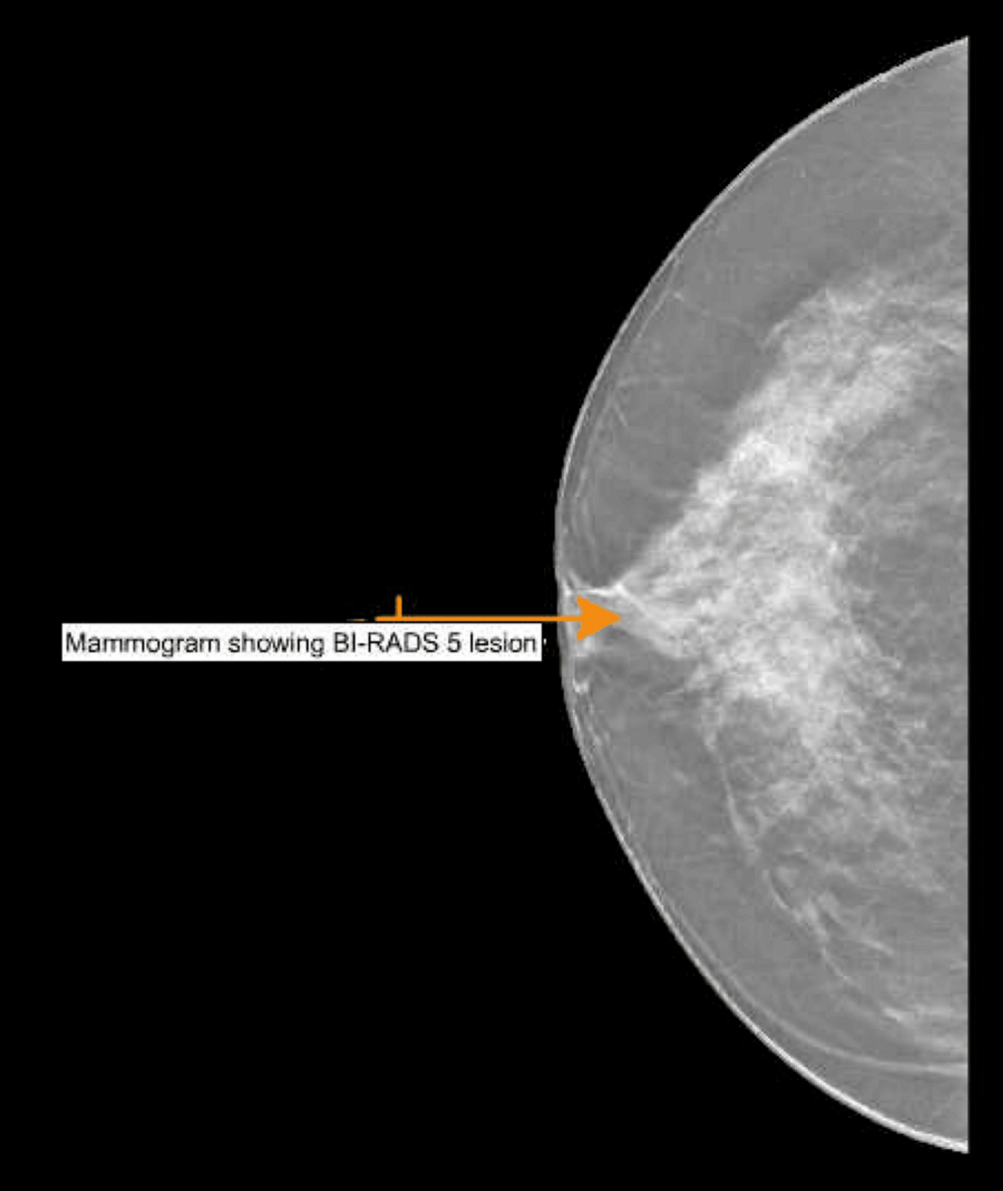
Mammogram of the patient showing a BI-RADS 5 lesion BI-RADS: Breast Imaging Reporting and Data System

**Figure 2:**
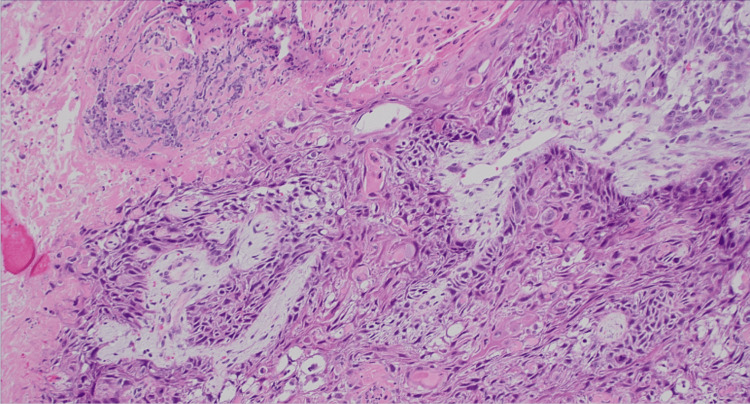
Histopathology slide showing squamous cell features including abundant eosinophilic cytoplasm, keratin pearls, and intercellular bridges

A 61-year-old woman with past medical history of essential hypertension, hyperlipidemia, and rotator cuff syndrome initially presented for the evaluation of a left breast lump associated with nipple inversion. It was first noticed by the patient almost two weeks prior to presentation. She did mention discomfort when lying on the affected side but did not endorse any pain or other systemic symptoms. Breast ultrasound revealed that there was a 26 × 27 × 18 mm hypoechoic lesion that was categorized as BI-RADS (Breast Imaging Reporting and Data System) 5 on mammogram (Figure 1). Of note, the patient did not have any other concerning lesions on her body, and recent imaging and lab work were also not concerning for any other malignancy. The patient underwent an ultrasound-guided core biopsy of the left breast lump. Results of the biopsy revealed grade 2 SCC with triple-negative immunophenotype and a Ki-67 proliferation index of approximately 90. The pathology slides (Figure 2) were also sent for review, and the diagnosis remained the same.

**Figure 3:**
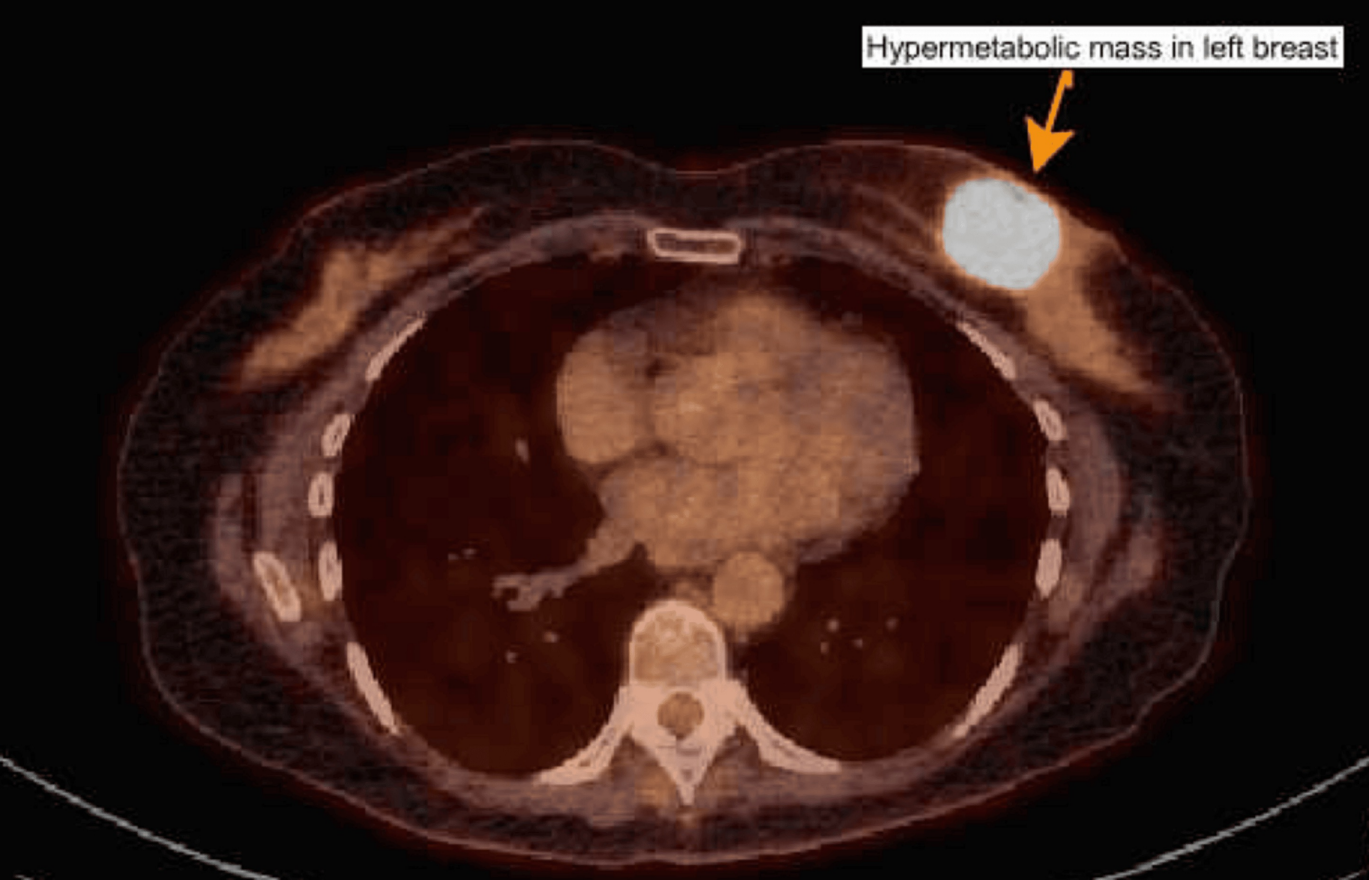
PET-CT showing a hypermetabolic mass in the left breast PET-CT: positron emission tomography/computed tomography

The whole-body positron emission tomography/computed tomography (PET-CT) revealed that the left breast mass was hypermetabolic (Figure 3). Additionally, it showed metabolically active lymph nodes in the left axilla. But it did not reveal any distant metastasis. The bone scan also did not show any metastatic lesions in the bones. After multidisciplinary consultation, surgery was recommended due to concern that neoadjuvant chemotherapy might not be very effective. The patient decided to undergo a bilateral mastectomy. Pathologic examination revealed no changes concerning for malignancy in the right breast. However, the specimen from the left breast revealed 42 × 38 × 36 mm, grade 3 metaplastic SCC with an extensive negative surgical margin. Seventeen axillary lymph nodes were analyzed, which did not show any evidence of metastatic disease. The results of the immunohistochemical analysis revealed that the tumor was triple-negative (ER -, PR -, HER-2-). The Ki-67 proliferation index was noted to be approximately 70%. Tumor cells showed strong and diffuse positivity for p40, confirming squamous differentiation. PD-L1 expression was positive, with a combined positive score of 80. After surgery, the patient received adjuvant systemic therapy in the form of combination chemotherapy and immunotherapy. The chemotherapy regimen consisted of Adriamycin (doxorubicin) and cyclophosphamide, followed by paclitaxel and carboplatin. In terms of immunotherapy, pembrolizumab was part of the regimen as well. This chemotherapy regimen was selected based on therapeutic strategies commonly employed for high-risk triple-negative and metaplastic breast carcinomas, as well as evidence suggesting potential activity of platinum-based agents in tumors with squamous differentiation. The immunotherapy agent pembrolizumab was incorporated into the regimen due to the tumor’s triple-negative phenotype and PD-L1 positivity.

The patient completed planned cycles of chemotherapy and was initially planned to continue pembrolizumab as maintenance therapy. However, she developed immune-mediated hypoadrenalism induced by pembrolizumab. Therefore, immunotherapy was discontinued. She also received appropriate endocrine replacement therapy for hypoadrenalism. PET-CT was repeated after completion of treatment, which did not reveal any evidence of the disease process.

## Discussion

Primary SCC of the breast is a very rare breast cancer, with an occurrence rate of less than 0.1% of all breast cancer [1-3]. It is believed to be a form of metaplastic breast cancer, and it is histologically different from the traditional invasive ductal carcinoma. Diagnosis of SCC of the breast requires ruling out metastatic squamous carcinoma of other primary areas in addition to ruling out direct extension of cutaneous or nipple squamous epithelium [2,4]. In our case, a primary breast origin was established by means of extensive imaging and clinical evaluation.

Pathogenesis and histogenesis

The pathogenesis of primary breast SCC is not fully understood. One of the theories suggests SCC of the breast arises from squamous metaplasia of ductal epithelial cells or pre-existing adenocarcinoma [5]. Alternatively, there are some authors who suggest de novo malignant transformation of pluripotent mammary stem cells [6]. The high and diffuse levels of p40, as noted in our case, are indicators of squamous differentiation, which is an important diagnostic feature [7].

Clinical and radiologic characteristics

Primary SCC of the breast presents clinically as a rapidly growing palpable mass and is often greater in size at the time of diagnosis as compared to invasive ductal carcinoma [3,8]. Nipple inversion and discomfort are often reported in these patients, but these are non-specific. Radiologic findings are variable, and there are no pathognomonic features characteristic of this malignancy; lesions may appear solid or cystic on ultrasound, often mimicking benign or inflammatory processes [8]. Therefore, the preoperative diagnosis is challenging, and histologic confirmation is a necessity.

Immunophenotype and molecular features

Most cases of SCC of the breast tend to have a triple-negative immunophenotype, which limits the potential options for targeted treatments [3,9]. High Ki-67 indices are commonly seen in this subcategory of breast carcinoma and are indicative of aggressive tumor biology. There is more and more evidence to suggest that metaplastic breast cancer including squamous cell cancer tends to express PD-L1 and contain conspicuous tumor-infiltrating lymphocytes, suggesting an immunogenic tumor microenvironment [10,11].

Treatment strategies

As the tumor is rare, there are no standardized treatment guidelines. The management usually entails surgical resection, and mastectomy is often undertaken due to the large size and central location of the tumor [4,9]. Involvement of axillary lymph nodes is also variable and might be less common than anticipated based on tumor size [3]. Of note, our patient also did not have lymph node involvement.

The effectiveness of conventional chemotherapy regimens is debatable. Some evidence suggests that there is inadequate responsiveness to anthracycline- and taxane-based therapy, especially in the neoadjuvant context [12]. There have been some reports of modest effectiveness of platinum-based regimens, which are often used in squamous carcinomas of other anatomic locations [9,12]. Addition of immunotherapy in triple-negative and metaplastic breast cancer has been promising, but high-quality evidence is lacking [11,13]. Radiation therapy is usually considered in an adjuvant situation in cases of large tumors or tumors with high-risk features, but evidence regarding the radiosensitivity of breast SCC is inconclusive [8,9].

Prognosis

The prognosis of primary breast SCC is variable. Some studies report a poorer prognosis as compared to invasive ductal carcinoma, with an increased likelihood of local recurrence as well as distant metastasis [3,14-16]. There are other studies that report survival rates comparable to invasive ductal carcinoma when adjusted for stage and completeness of resection [4,17]. The most important prognostic factors appear to be tumor size, grade, and stage at the time of diagnosis (Table 1).

**Table 1:** Summary of selected published studies on primary squamous cell carcinoma of the breast

Study	Year	Chemotherapy regimen	Treatment setting	Reported outcome
Cardoso et al. [1]	2000	CMF (cyclophosphamide, methotrexate, fluorouracil) or anthracycline-based regimens	Adjuvant	Variable responses; some cases developed recurrence
Macia et al. [18]	2006	Anthracycline-based chemotherapy	Adjuvant	Limited response reported in several cases reviewed
Aparicio et al. [15]	2008	Anthracycline-based chemotherapy	Adjuvant	Mixed outcomes; benefit of chemotherapy uncertain
Flikweert et al. [17]	2008	Anthracycline followed by taxane	Adjuvant	Reported disease-free survival after multimodal treatment
Bhosale et al. [19]	2013	Cyclophosphamide, doxorubicin, and 5-fluorouracil	Adjuvant	Favorable short-term outcome
Behranwala et al. [14]	2003	Anthracycline-based chemotherapy	Adjuvant	Limited effectiveness reported in some cases
Stevenson et al. [4]	1996	CMF regimen	Adjuvant	Variable outcomes with occasional recurrence
Weigel et al. [16]	1996	No chemotherapy administered	-	Managed with surgery alone

## Conclusions

Primary SCC of the breast is a rather rare and aggressive cancer with specific clinicopathologic features. The diagnosis of primary SCC of the breast requires exclusion of other primary SCC malignancies and is confirmed through biopsy and immunohistochemistry. Surgical resection remains an important part of management, and systemic therapy is still evolving. This case highlights the potential of immunotherapy use in PD-L1-positive primary SCC disease patients. There is a need for further research to establish the best management strategies for this rare type of breast cancer.
